# Fuzzy Evaluation of Crowd Safety Based on Pedestrians’ Number and Distribution Entropy

**DOI:** 10.3390/e22080832

**Published:** 2020-07-30

**Authors:** Xuguang Zhang, Qinan Yu, Yuxi Wang

**Affiliations:** School of Communication Engineering, Hangzhou Dianzi University, Hangzhou 310000, China; qinanyu536@163.com (Q.Y.); yxwang@hdu.edu.cn (Y.W.)

**Keywords:** crowd video monitoring, crowd safety evaluation, distribution entropy, fuzzy inference

## Abstract

Crowd video monitoring and analysis is a hot topic in computer vision and public management. The pre-evaluation of crowd safety is beneficial to the prediction of crowd status to avoid the occurrence of catastrophic events. This paper proposes a method to evaluate crowd safety based on fuzzy inference. Pedestrian’s number and distribution uniformity are considered in a fuzzy inference system as two kinds of attributes of a crowd. Firstly, the pedestrian’s number is estimated by the number of foreground pixels. Then, the distribution uniformity of a crowd is calculated using distribution entropy by dividing the monitoring scene into several small areas. Furthermore, through the fuzzy operation, the fuzzy system is constructed by using two input variables (pedestrian’s number and distribution entropy) and one output variable (crowd safety status). Finally, inference rules between the crowd safety state and the pedestrian’s number and distribution uniformity are constructed to obtain the pre-evaluation of the safety state of the crowd. Three video sequences extracted from different scenes are used in the experiment. Experimental results show that the proposed method can be used to evaluate the safety status of the crowd in a monitoring scene.

## 1. Introduction

Crowd behavior analysis is the application field of many disciplines such as artificial intelligence [1,2], safety management [3,4], and computer vision [5,6]. There are three kinds of research methods for crowd behavior analysis, i.e., controlled experiment [7,8], crowd simulation [9,10], and crowd video monitoring [11,12]. As video monitoring can obtain the information of the scene, and monitor, alarm, record, and query the crowd status, crowd video monitoring plays an important role in the field of crowd behavior analysis. Many contributions have been proposed for crowd video monitoring such as target detection and tracking [13,14], crowd counting and density estimation [15,16], and crowd abnormal behavior detection [17,18]. However, traditional crowd video monitoring methods are often limited in the field of crowd counting and other feature detection, or to detect the abnormal and disaster events that have occurred. These methods neglect to evaluate the movement state of the crowd before catastrophic events. In fact, it is more valuable for crowd safety management to reduce the probability of catastrophic events by evaluating the safety status of the crowd.

**Figure 1:**
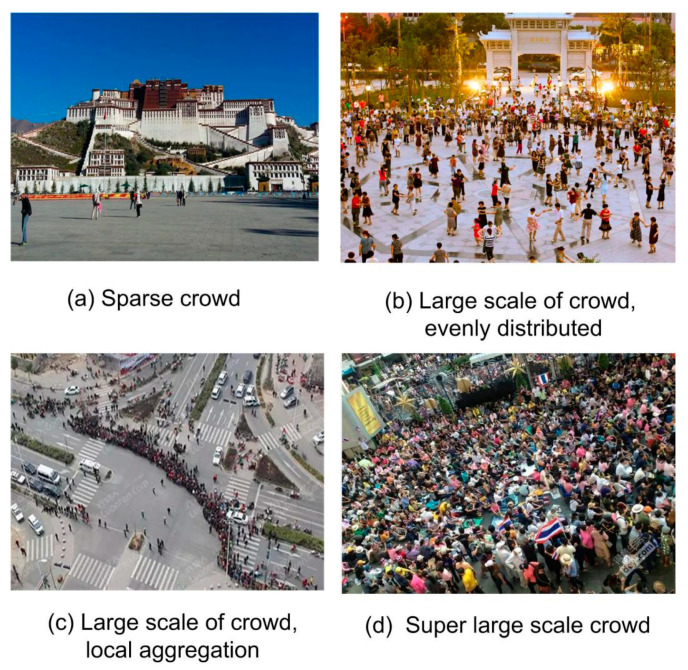
The safety status of different crowd sizes and distribution uniformities.

The risk of crowd movement can usually be reflected by attributes such as number of pedestrians and distribution uniformity, and is unsuitable for evaluation by one attribute alone. For example, a sparse crowd is safer (Figure 1a), and a crowd with a super large scale is more likely to have dangerous behaviors (Figure 1d), while the status with a middle or large-scale crowd cannot be evaluated only by the number of pedestrians. If the crowd is evenly distributed, it is safer (Figure 1b), but if the pedestrians gather in a certain area, there may be abnormalities (Figure 1c). Therefore, before the occurrence of destructive events, it is very important to calculate and integrate the number of pedestrians and the uniformity of distribution to evaluate the safety status of the crowd.

In this paper, the number of pedestrians and the distribution uniformity of a crowd are extracted to distribute the safety status of the crowd. To calculate the number of pedestrians in a crowd, there are two kinds of methods. One is based on feature extraction and regression, the other is based on deep learning [19,20]. Deep learning has shown good performance in crowd counting. In this work, we only take the number of pedestrians as the input of the fuzzy reasoning system, rather than to report the exact number value. In order to enhance the efficiency of the method and improve the practicability of the method, we choose the way of feature description to express the number of pedestrians. We choose the number of foreground pixels as the feature to describe the number of pedestrians. At the same time, we propose a method based on the theory of Shannon entropy to express the distribution uniformity of a crowd. We divide the monitoring scene into several areas, and calculate the crowd distribution entropy according to the distribution probability of the number of pedestrians or foreground pixels in each area, to measure the uneven degree of crowd distribution. Shannon entropy is a classical and effective method to measure the uncertainty of information [21,22,23]. Shannon entropy has been used in many fields such as interpreting the information in atomic states expressed for a Ni-like isoelectronic sequence [24], measuring the characteristics of two-dimensional fractional Brownian fields [25], and encrypting an image based on entropy analysis and a chaotic system [26]. After obtaining the two crowd attributes, i.e., the number of pedestrians and the distribution uniformity of a crowd, the mapping rules between crowd attributes and safety status can be established to evaluate the safety status of the crowd. However, the description of crowd attributes is usually imprecise, such as the number of pedestrians being large or small, or the crowd distribution being uniform or convergence. Fuzzy theory brings solutions to the uncertainty of description. In 1965, Zadeh published the first paper on fuzzy sets [27], and then many extended theories came into being. For example, Mamdani further discussed the method of fuzzy reasoning [28]. Fuzzy inference mainly includes the fuzziness of variables, the output of rule-based inference, and the defuzziness. Many contributions have been proposed using the fuzzy system, such as controlling nonlinear systems using fuzzy logic based on the Observer [29], and merging the Takagi-Sugeno fuzzy model into a broad learning system [30]. In this paper, according to the fuzzy framework, we fuzzy the two attributes of the number of pedestrians and the distribution uniformity of a crowd, and establish inference rules according to the relationship between crowd attribute and safety status, to comprehensively evaluate the current crowd safety status.

The rest of the paper is organized as follows: Section 2 is the related work. Section 3 introduces the calculation of the crowd attribute of pedestrian’s number. Section 4 introduces the measurement method of crowd uniformity based on distribution entropy. The fuzzy inference system of crowd safety will be given in Section 5. Section 6 presents the experimental results on different video sequences. Section 7 summarizes the paper.

## 2. Related Work

### 2.1. Crowd Behavior Models

A crowd is a complex self-organizing system. Constructing a crowd behavior model is helpful to understand crowd behavior. Many contributions have been proposed to describe crowd behavior using different models. The first kind of method mainly studies crowd behavior based on a psychological and social basis, such as the influence of reward structure on crowd behavior [31], and the influence of social relations among individuals on panic behavior [32]. Then, a molecular-based method has been proposed for describing crowd motion. One of the most famous is the social force model [33,34]. Psychological and physical forces are fused to describe crowd behavior in the social force model. The social force model has been used to simulate crowd panic status [35]. In recent years, models based on agent and probabilistic models have been proposed, which can help to express the interaction between people, such as simulating crowd motion on a realistic physical space [36], simulating crowd turbulence using inter-personal stress and acceleration [37], structuring an agent model for long-range collision avoidance [38], simulating heterogeneous crowd motion based on personality trait theory [39], and using the probability of attraction or repulsion agents to simulate the behavior of emergency crowd [40].

### 2.2. Crowd Status Detection

Although the pre-assessment of crowd safety status has important value, current research on the video monitoring of crowd status is more concentrated in the field of crowd anomaly detection. There are two kinds of crowd abnormal behavior, i.e., global abnormal and local abnormal. Global anomalies usually detect the panic and chaos of the crowd as a whole, such as using energy and entropy to detect crowd abnormal behavior [41], and using a sparse expression method for detecting abnormal crowd behavior [12]. Local anomalies usually focus on pedestrians or vehicles that are different from most pedestrians in the scene. For example, the local abnormal behavior is detected by extracting the track of pedestrians in [42], and the anomaly in the scene is located by analyzing the texture features in the image in [43].

## 3. Calculation of the Pedestrian’s Number Attribute

To gain the crowd attribute of the pedestrian’s number, we calculate the foreground pixels in each frame. Therefore, the pedestrian’s number can be gained using the least squares estimation according to the relationship between the number of foreground pixels and pedestrians.

### 3.1. Foreground Pixel Extraction

In each scene, the background image should be calculated using the average value of a series of images in the scene over a period. The foreground image can be gained using a threshold by comparing the current frame and the background image, as formula (1) shows:

(1) $$output(x,y)=\{\begin{array}{cc} 1, & \left|I_{t}(x,y)-\bar{I}(x,y)\right|>threshold\\ 0, & otherwise \end{array}$$

where *output(x,y)* is the foreground image, *I_t_(x,y)* is the current image, and $\bar{I}\left(x,y\right)$ is the background image.

In order to reduce the holes and noise in the foreground image extracted by the method of background difference, morphological operation is used to deal with them in this work.

### 3.2. Pixel Interpolation

Due to the different distances from the camera, the size of pedestrians in the image will change, which will affect the accuracy of the estimation of the number of pedestrians. We use the method in reference [44] to assign different weights to different positions of the image to weaken the influence of the distance between the pedestrians and the camera.

**Figure 2:**
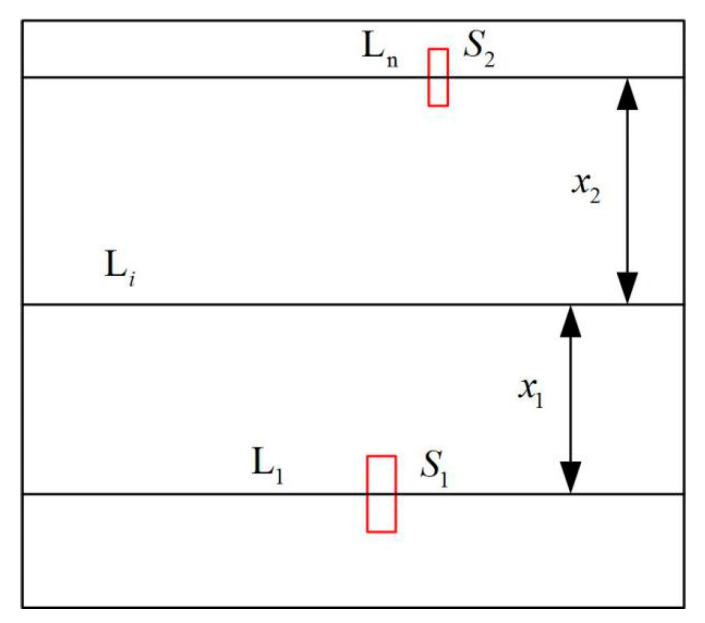
The principle of foreground pixel weight interpolation.

The interpolation principle of weight is shown in Figure 2. For a specific scene, we select the furthest and nearest pedestrian from the camera as the reference target and calculate the foreground pixel areas occupied by the pedestrians, which is calculated according to the following formula:

(2) $$S=\sum_{i=1}^{w}\sum_{j=1}^{h}F_{ij}$$

where *w* and *h* represent the width and height of the image, respectively. *F_i,j_* is set as 1 if the pixel is foreground, and *F_i,j_* is set as 0 if the pixel is background.

Draw a horizontal line as the reference line at the center of mass of two reference pedestrians. Record the reference line near the camera as *L*_1_ and the reference line far from the camera as *L_n_.* The change rate of the reference pedestrian’s area at *L_n_* and *L*_1_ is as follows:

(3) $$R=\frac{S_{2}}{S_{1}}$$

where *S*_1_ and *S*_2_ represent the pixel area of the pedestrians nearest and farthest from the camera, respectively. The weight of the pixel on the reference line *L*_1_ is set as *w*_1_ = 1. The weight of the pixel on the reference line *L_n_* is set as *w_n_* = 1/*R*. If the distance between the point on any line *L_i_* (0 ≤ *I* ≤ *H*) and the reference line *L*_1_ and *L_n_* is *x*_1_ and *x*_2_, respectively, the weight of the pixel on the line *L_i_* can be obtained by the linear interpolation method:

(4) $$w_{i}=w_{1}+\frac{(w_{n}-w_{1})\times x_{1}}{x_{1}+x_{2}}=\frac{w_{1}+\frac{x_{1}}{x_{2}}w_{n}}{1+\frac{x_{1}}{x_{2}}}=\frac{x_{2}\times R+x_{1}}{R\times (x_{1}+x_{2})}$$

Therefore, the foreground pixel area of pedestrians at the *L_i_* position in the weighted image can be expressed as:

(5) $$S=\sum_{i=1}^{M}\sum_{j=1}^{N}F_{ij}\times w_{i}$$

### 3.3. Pedestrian’s Number Regression

**Figure 3:**
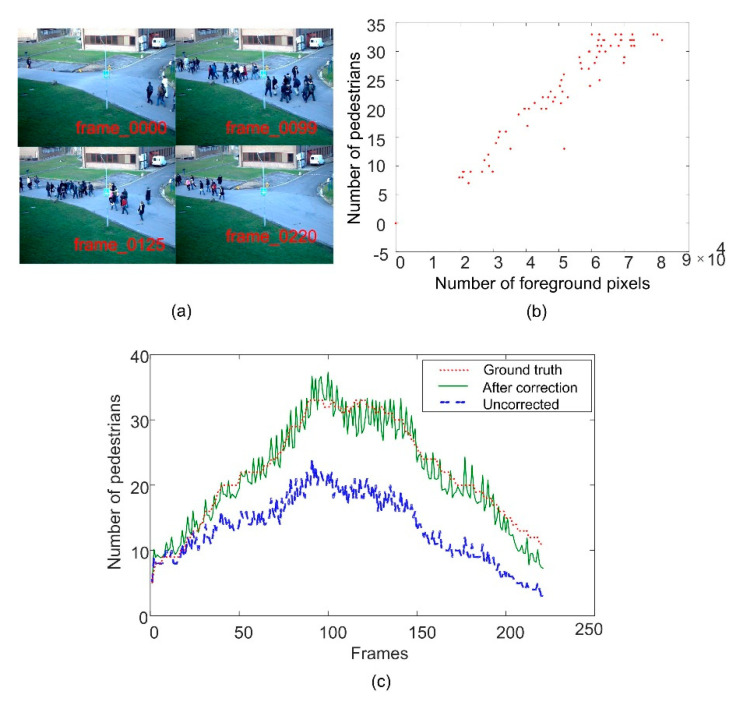
Examples of crowd count results. (**a**) Sample frame images; (**b**) the relationship between the number of pedestrians and foreground pixels; (**c**) crowd count results before and after the weighted operation.

It is a traditional and effective method to use a regression strategy to count the pedestrians. The main idea of this method is to establish a mapping relationship from feature to pedestrian number [45]. The number of pedestrians is estimated by characteristics. In this paper, the least squares method is used to regress the crowd number. That is to say, a linear relationship is used to express the number of pedestrians and foreground pixels. This linear relationship can be described as:

(6) $$Ped_{num}=k_{1}S+b_{1}$$

where *k*_1_ and *b*_1_ are the parameters to be determined, $Ped_{num}$ represents the number of pedestrians, and *S* represents the number of foreground pixels in the image. The effect of crowd counting is verified by using the time_13-57_view_001 sequence in the pets2009 dataset, and the results are shown in Figure 3. Figure 3b shows an approximate linear relationship between the number of pedestrians and foreground pixels. Figure 3c shows the crowd count results before and after the weighted operation. It can be seen that the count results after the weighted operation are more accurate.

## 4. Describing Crowd Uniformity Using Distribution Entropy

In some scene, the number of pedestrians is almost constant. The crowd safety status cannot be judged only using the number of pedestrians. The uniformity of the distribution of a crowd will play a role in these scenes. In this paper, we propose a distribution entropy to measure the uniformity of the distribution of a crowd.

### 4.1. Regional Division of Crowd Movement

**Figure 4:**
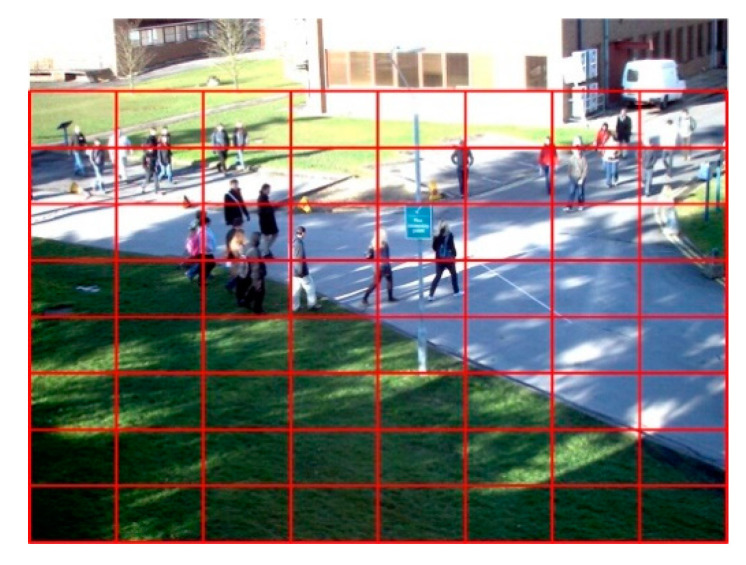
Regional division of crowd monitor scene.

To measure the uniformity of crowd distribution, the monitor image should be divided into several boxes. In the monitoring scene, we divide the area into several uniform blocks. It is worth mentioning that there will be some areas without pedestrians in the image. We will remove this area and then divide the scene image. As shown in Figure 4, some areas above the image are considered invalid. For each divided image area, the number of pedestrians will be calculated using the method in Section 3. As there is a linear relationship between the number of pedestrians and the number of foreground pixels, in some scenes, we can also use the number of foreground pixels to approximately replace the number of pedestrians.

Suppose that the foreground image extracted at frame *i* is divided into *m* blocks, and *S_i_* represents the area of the foreground pixels of the whole foreground image.

(7) $$S_{i}=\sum_{j=1}^{m}S_{ij}$$

where S*_ij_* represents the area occupied by the *j*th small block foreground in the *i*th image. The ratio of the number of foreground pixels in this region to the area of the foreground in the whole image is:

(8) $$R_{ij}=\frac{S_{ij}}{S_{i}}$$

### 4.2. Calculation of Crowd Distribution Entropy

**Figure 5:**
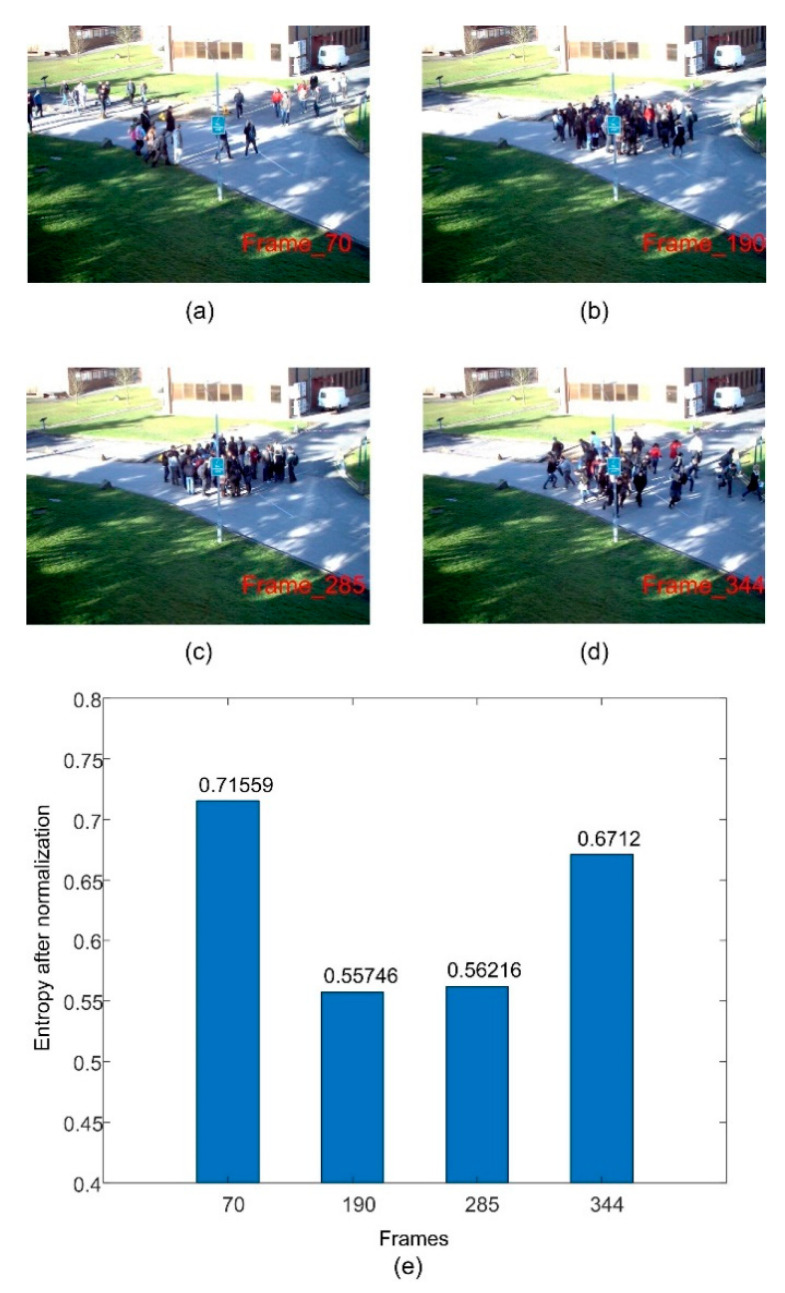
Crowd distribution entropy of different sample frames. (**a**–**d**) are the sample frame images; (**e**) is the result of the distribution entropy of the four images.

For a monitor scene, because the monitoring scene is divided into several areas, the probability of pedestrians appearing in a certain area is uncertain. The uniformity of pedestrian distribution in the scene can be measured according to this uncertainty. For example, if pedestrians are evenly distributed in the scene, the probability of pedestrians appearing in each sub area is equal. If pedestrians gather in a certain area, the probability of pedestrians appearing in that area increases, and the probability of other areas decreases. Shannon entropy is a classical and effective method to measure the uncertainty of information. In this paper, we use Shannon entropy to measure the uniformity of crowd distribution. The entropy of crowd distribution can be calculated as:

(9) $$E_{i}=-\left(\overset{m}{\underset{j=1}{\sum }}R_{ij}logR_{ij}\right)$$

where *R_ij_* is the probability of pedestrians appearing in area *j* of frame *i*. In the case of the same number of pedestrians, the larger the entropy, the more uniform the distribution of pedestrians in the scene; and the smaller the entropy, the more concentrated the pedestrians are in the scene. Figure 5 shows an example where the distribution entropy changes significantly when the crowd gathers and disperses.

## 5. Fuzzy Inference and Evaluation of Crowd Safety Status

The crowd safety status can be estimated using the crowd attribute. In this paper, we use two characteristics of pedestrians’ number and distribution uniformity to describe crowd safety. However, the description of these two characteristics is uncertain. To make better use of crowd characteristics, we designed a fuzzy inference system to boost the performance of crowd safety status estimation.

### 5.1. Fuzzification

In this paper, there are two input variables (pedestrians’ number and distribution entropy) and one output variable (safety state) in the fuzzy system. In the fuzzy inference system, the first step is fuzzification. In this paper, each variable is quantized into five levels.

#### 5.1.1. Fuzziness of Pedestrians’ Number

**Figure 6:**
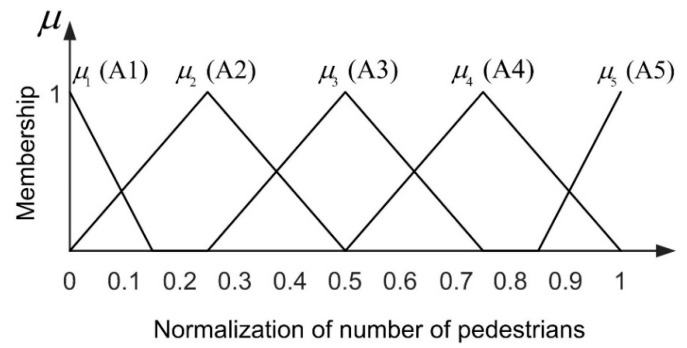
Membership function of pedestrians’ number.

In this work, the number of pedestrians in the scene is normalized to between 0 and 1 by dividing the maximum number of pedestrians with which the scene can be accommodated. The maximum number of pedestrians that can be accommodated in different scenes is different, and in some places, such as in the area of buildings, it is impossible for pedestrians to walk. Therefore, we calculate the number of pedestrians that can be accommodated in the scene according to the movement energy accumulation of pedestrians. Moving energy acquisition accumulates the foreground extracted image in the whole sequence and then retains the area of pedestrian presence through threshold filtering. According to the strategy adopted in the contribution [46], the pedestrian number is divided into five grades (A1: Very few, A2: Few, A3: Medium, A4: Many, A5: A great many) according to the proportion of the pedestrians to the maximum capacity. According to the five grades and the simple triangle model, we design the membership function of pedestrians’ number, which can be seen in Figure 6.

#### 5.1.2. Fuzziness of Entropy of Crowd Distribution

**Figure 7:**
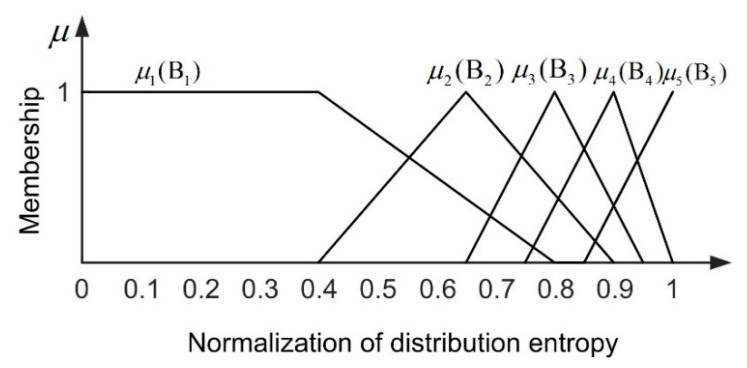
Membership function of uniformity of crowd distribution.

The uniformity of crowd distribution is expressed by crowd distribution entropy. The entropy of crowd distribution is normalized from 0 to 1. The theoretical maximum entropy in the scene is determined by the number of blocks divided. In this paper, the variable of crowd distribution uniformity in the scene can be divided into five levels (B1: Very uneven, B2: Uneven, B3: Medium, B4: Even, B5: Very even). According to the triangle function, we establish the membership function of the crowd distribution uniformity, as shown in Figure 7. The membership degree of distribution entropy is not uniform. This is related to the change trend of the distribution entropy. The membership division of the distribution entropy is close to the trend of the entropy.

#### 5.1.3. Fuzziness of Crowd Safety Status

**Figure 8:**
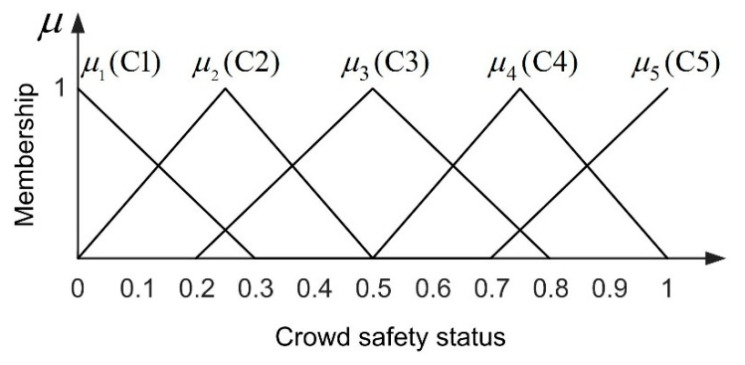
Membership function of crowd safety status.

The output variable of the fuzzy system in this paper is crowd safety status. Here, we assume that the greater the crowd safety value, the less dangerous the scene. On the contrary, the smaller the crowd safety, the greater the risk of the scene. We normalized the range of crowd safety values to between 0 and 1. Therefore, the safety value of the crowd in the scene can be divided into five levels (C1: Very dangerous, C2: Dangerous, C3: Medium, C4: Safe, C5: Very safe). In order to simplify the calculation, we also use the triangle function to build the membership function of the crowd safety state, as shown in Figure 8.

### 5.2. Fuzzy Inference

In order for the fuzzy system to make an effective decision, it is very important to make reasonable inference rules. Our system takes the relationship between crowd safety and crowd attributions into account with the following principles conformed.

(1) The lower the number of pedestrians in the crowd, the safer the state of the crowd. On the contrary, the higher the number of pedestrians, the stronger the potential risk of the crowd.

(2) The more evenly distributed the crowd, the higher the safety of the crowd. On the contrary, the more uneven the crowd distribution, the stronger the potential risk of the crowd.

According to the principles above, we designed 25 rules to infer the safety state of the crowd. These rules are listed in the “fuzzy inference table” (see Table 1). For example, the first rule in Table 1 can be described as: IF “the uniformity of crowd distribution is very uneven” AND “pedestrian number is very few,” THEN “the crowd safety status is very safe.”

### 5.3. Defuzzification

Through the fuzzy inference system, we obtain the fuzzy solution of the crowd safety status. Hence, defuzzification should be used to precisely quantify the crowd safety status. We used the method of “center of gravity” to defuzzify the output crowd safety status for each fuzzy decision. It mainly takes the center of gravity of the area enclosed by the curve of the membership function and the abscissa. The calculation process of the center of gravity method is shown in formula (10):

(10) $$z=\frac{\sum_{i=1}^{N}\left(z_{i}\mu_{C^{*}}(z_{i})\right)}{\sum_{i=1}^{N}\left(\mu_{C^{*}}(z_{i})\right)}$$

where *z* is the crisp output, *N* is the number of rules, and $\mu_{C^{*}}\left(z_{i}\right)$ is the membership function.

## 6. Experiment and Discussion

In this section, we analyze and discuss the experimental results of crowd safety status evaluation. Three sequences from different scenes are used in this work. One is the crowd movement simulated sequence composed by unity 3D software. One is the view-001 sequence in time 14-33 in the Pets2009 database. The other one is the WorldExpo’10 database train-video-100736-squap1-04-s20100626083000000e201006263059000-new.split.319-2 scene (hereinafter referred to as 100736). There are 42 videos of this scene in this dataset. One frame is extracted every 10 frames to form a sequence for the experiment. The manual measurement values of the safety status of the crowd was manually marked by 10 volunteers and calculated by average. The frame numbers and image sizes of the video sequences are shown in Table 2. The experimental platform is a PC with a 2.80 GHz processor and 16 GB memory.

**Figure 9:**
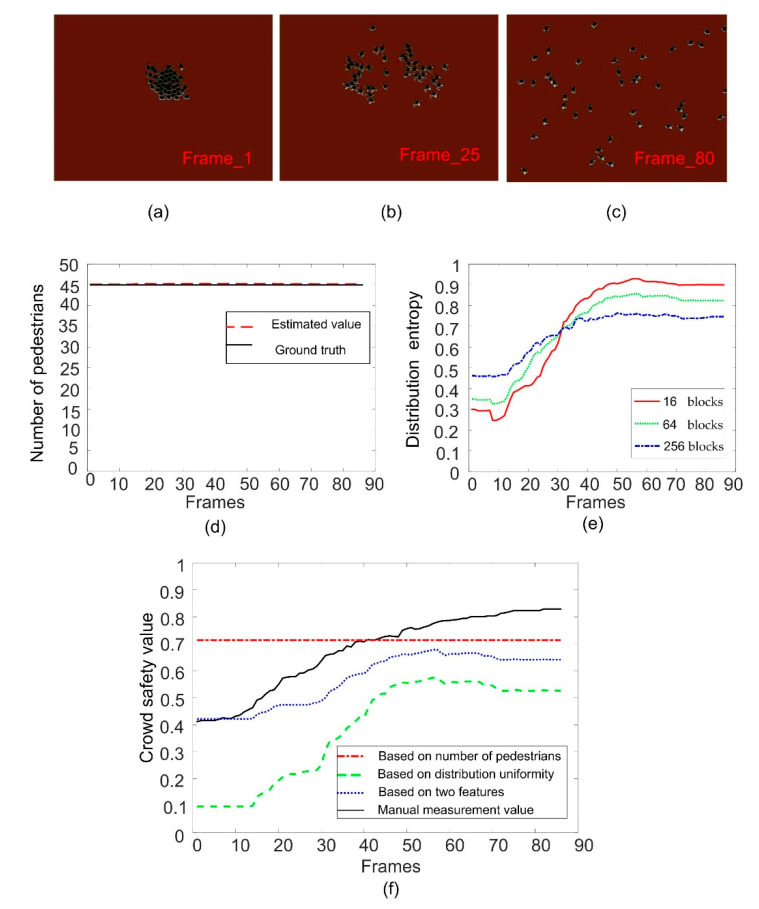
Crowd safety status evaluation for unity 3D sequence. (**a**–**c**) are the sample frame images; (**d**) is the result of crowd counting; (**e**) is the curve of crowd distribution entropy; (**f**) is the results of crowd safety evaluation.

**Figure 10:**
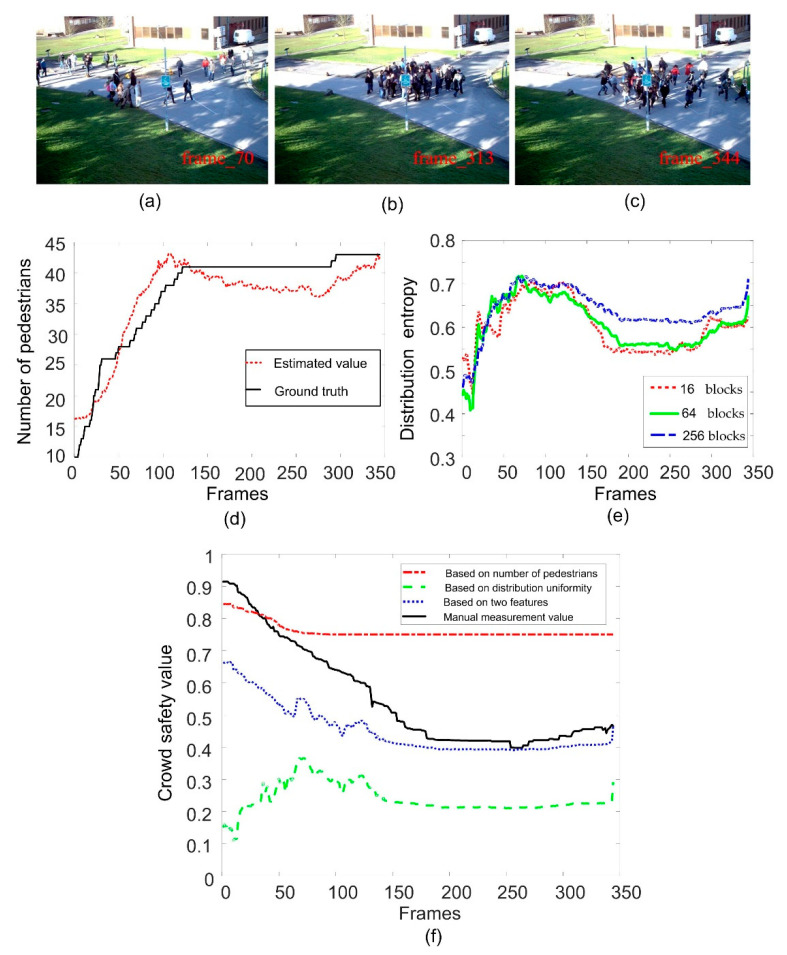
Crowd safety status evaluation for view001 sequence. (**a**–**c**) are the sample frame images; (**d**) is the result of crowd counting; (**e**) is the curve of crowd distribution entropy; (**f**) is the results of crowd safety evaluation.

**Figure 11:**
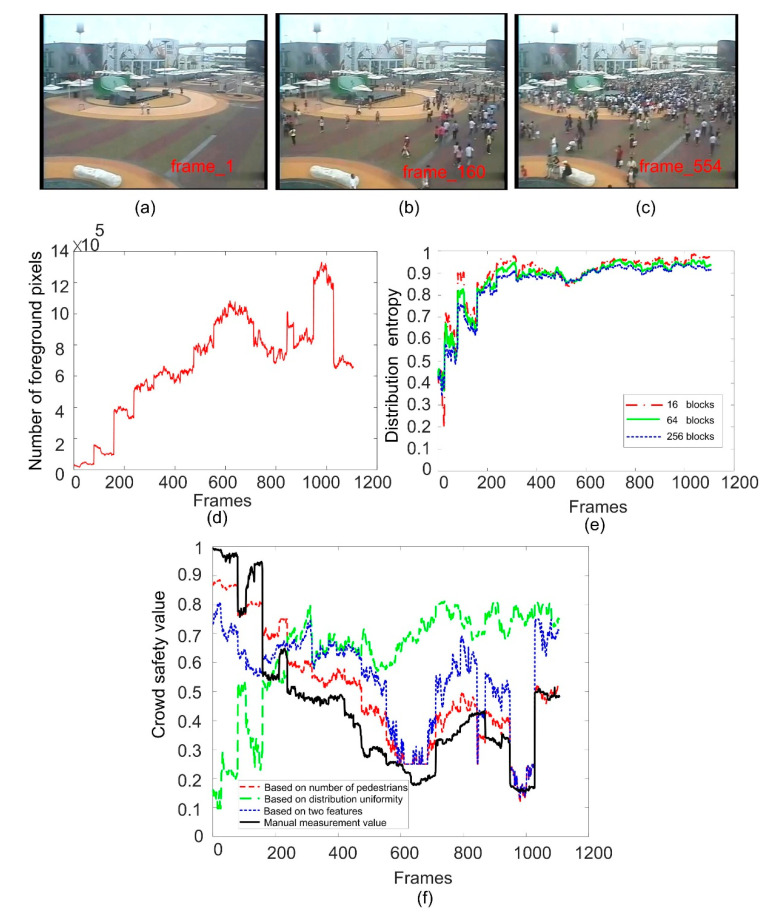
Crowd safety status evaluation for 100736 sequence. (**a**–**c**) are the sample frame images; (**d**) is the curve of the number of foreground pixels; (**e**) is the curve of crowd distribution entropy; (**f**) is the results of crowd safety evaluation.

In order to evaluate the effect of image block size on the calculation of distribution entropy, we compare the distribution entropy of images divided into different blocks. In Figure 9e, Figure 10e and Figure 11e, we give the results of calculating the distribution entropy with 16 blocks, 64 blocks, and 256 blocks. It can be seen that the fluctuation is larger when the image is divided into 16 blocks, and it is more stable when the image is divided into 256 blocks. Too few blocks will be too sensitive for the crowd distribution, and too many blocks will make it difficult to reflect the change in crowd uniformity. In this study, 64 blocks were selected as the number of blocks. When the distribution entropy is used to measure the crowd distribution uniformity, it will be affected by the camera angle. That is to say, the real scene area occupied by same-size blocks will be different, and the number of pedestrians will also be different. Of course, this effect can be solved by dividing the image blocks into different sizes according to the distance from the camera. Of course, this problem can be divided into specific scenes in practical application.

### 6.1. Experimental Results of Safety Status Evaluation

We use three video sequences to verify the validity of fuzzy inference, and compare the results of inference with two features and one single feature. The first sequence is video simulated by unity 3D. The results can be seen in Figure 9. Figure 9a–c show the sample frames of different crowd states. Figure 9d shows the estimated number of pedestrians. It can be seen that the number of pedestrians in this scene is 45 and remains unchanged. Figure 9e shows the curve of crowd distribution entropy. It can be seen the change in entropy value coincides with the process of crowd aggregation and dispersion. Figure 9f shows the comparison of the results of safety evaluation based on the different characteristics of the crowd. It can be seen that the safety state evaluation based on the number of pedestrians does not change, and the whole sequence is in a safe state, which cannot reflect the change in the state of the crowd. Using only the uniformity of crowd distribution to infer the safety state of the crowd can reflect the change in crowd state, but the result is too extreme because there is no restriction in the pedestrians’ number characteristic. However, the evaluation of crowd safety state combined with the two characteristics of pedestrians’ number and distribution uniformity is closer to the manual measurement value.

The second video is the Time_14-33/View_001 sequence from the Pets2009 dataset. Figure 10a–c show the sample frames of different crowd states. We estimate the number of pedestrians in the crowd by the number of foreground pixels, as shown in Figure 10d. The estimated result can reflect the change in the number of pedestrians and is close to the ground truth. We can see that the number of pedestrians in the scene increases continuously at first, and then does not change much. The overall growth in the number of pedestrians is not very large. If the safety fuzzy evaluation is based on the number of pedestrians, the safety value of the crowd does not change dramatically, as shown by the red curve in Figure 10f. Figure 10e shows the change curve of the entropy of crowd distribution. We can see that the crowd distribution entropy can reflect the change in crowd aggregation and dispersion, but when the number of pedestrians is small, it will be mistaken for aggregation. Therefore, it is not ideal to use entropy of crowd distribution alone to infer the safety state. If the number of pedestrians and the distribution of the crowd are combined, the change curve of the crowd safety value will be closer to the trend of the manual measurement value, making up for the shortage of using a single number of pedestrians and distribution entropy, as shown by the blue curve in Figure 10f.

The third video sequence comes from the WorldExpo’10 database, which we call the 100736 sequence for short. In the database, the monitoring time of the scene is long, which can reflect the change in crowd state. We extract one frame from every 10 frames to form the experimental video sequence. Figure 11a–c show several sample frames. It can be seen that the number of pedestrians in the scene varies greatly. In Figure 11d, we show the change curve of the number of foreground pixels to reflect the change trend of the number of pedestrians in the scene. The number of foreground pixels here is weighted by the factor of view angle change. As there are too many pedestrians in the scene, it is difficult to accurately calculate the ground truth of the number of pedestrians, so in this sequence, we use foreground pixels to replace the number of pedestrians to infer the crowd safety. Figure 11e shows the change in crowd distribution entropy. As the size of the crowd is very large in the later stages of the scene, the crowd distribution entropy is relatively large, that is to say, the crowd distribution is relatively uniform, so it is difficult to reflect the change in crowd safety state by using distribution entropy alone. Therefore, in this scene, the characteristics of the number of pedestrians (foreground pixels) are more effective in inferring the safety status of the crowd in this sequence. The combination of the two features can also be used to predict the safety of the crowd. In this sequence, it is not as accurate as using the characteristics of the number of foreground pixels alone. On the whole, combining the two features to carry out crowd safety reasoning can take into account the advantages and disadvantages of these two features, making the adaptability of the evaluation of the safety status of the crowd in different scenes stronger.

### 6.2. Global Performance Analysis using MAE and MRE

In Section 6.1, we provide the prediction curve of crowd safety status. In this section, we calculate two parameters to evaluate the global performance of each method for the whole sequence. We calculated the MAE (mean absolute error) and MRE (mean relative error) parameters. Mean absolute error is the mean value of absolute error between the predicted value and manual measurement value, which is expressed by MAE. The mean relative error is used to represent the mean value of the relative error between the predicted value and the manual measurement value, which is represented by MRE. The MAE and MRE are calculated as follows:

(11) $$\mathrm{MAE}=\frac{1}{M}\sum_{i=1}^{M}\left|E\left(i\right)-T\left(i\right)\right|$$

(12) $$\mathrm{MRE}=\frac{1}{M}\sum_{i=1}^{M}\frac{\left|E\left(i\right)-T\left(i\right)\right|}{T\left(i\right)}$$

where *M* represents the frames number of the test sequence, and *E(i)* and *T(i)* represent the estimated value and the manual measurement value of the *i*th frame image, respectively. Table 3 compares the results of the proposed method (combining the characteristics of the number of pedestrians and distribution uniformity) to infer crowd safety with the results of using a single feature. From the MAE and MRE in the table, it can be seen that the method of combining the two features can achieve a lower error value in most experimental sequences, and has a wider range of adaptability in all the three sequences, while the single feature may have a better effect on some scenes, but the adaptability is poor, and it may cause great errors to other scenes.

In order to evaluate the practicability of the method, we calculated the running time of the method. The algorithm mainly consists of four parts: Foreground extraction, pedestrian’s number estimation, distribution entropy calculation, and fuzzy inference. Taking view001 sequence as an example, the average computing time of the whole algorithm is about 1.788 s per frame.

## 7. Conclusions

We propose an effective method to evaluate the crowd safety status using the pedestrians’ number and distribution entropy attributes. There are two contributions in this paper. One is that we propose a method to measure the uniformity of crowd distribution by Shannon entropy, the other is to construct a fuzzy system, which uses two attributes of pedestrians’ number and distribution entropy to infer the safety state of a crowd. The experimental results show that the proposed method can be used to evaluate the safety status of the crowd, and the fuzzy inference system combined with two attributes has a wider range of adaptability than a single attribute. Due to the complexity of crowd behavior, the method proposed in this paper is still inadequate. For example, the different speed of pedestrian movement will affect the safety of the crowd. The effect of using distribution entropy to describe crowd distribution can still be improved. In future work, we will try to combine the characteristics related to pedestrian speed or energy to depict more complex crowd behaviors. When describing the uniformity of crowd distribution, we will pay more attention to the distribution of the local image region to improve the calculation method of entropy to improve the performance of crowd distribution measurement. In addition, we will extract more crowd attributes and establish more appropriate rules to adapt to different scenes.

## Figures and Tables

**Table 1:** Fuzzy inference for crowd safety status.

Variables		Number of Pedestrians				
		*A1*	*A2*	*A3*	*A4*	*A5*
**Crowd Distribution Uniformity**	*B1*	*C5*	*C2*	*C2*	*C1*	*C1*
	*B2*	*C5*	*C3*	*C2*	*C1*	*C1*
	*B3*	*C5*	*C4*	*C3*	*C2*	*C1*
	*B4*	*C5*	*C4*	*C4*	*C2*	*C1*
	*B5*	*C5*	*C4*	*C4*	*C2*	*C1*

**Table 2:** Video data statistics of different scenes.

Video Sequences	Parameters	Values
View_001	Frame number	344
	Image size	768 × 576
100736	Frame number	1106
	Image size	720 × 352
Unity 3D	Frame number	86
	Image size	850 × 648

**Table 3:** Comparison of results using different crowd characteristics.

Video Sequences	Parameters	Number of Pedestrians	Distribution Uniformity	Combination of Two Features
Unity	MAE	0.1157	0.2890	0.1066
	MRE	0.2112	0.4655	0.1480
View_001	MAE	0.2166	0.3124	0.1005
	MRE	0.4750	0.5429	0.1568
100736	MAE	0.0815	0.3751	0.1688
	MRE	0.2143	1.1314	0.4141

## References

[1] Dong Z., Zhang R., Shao X., Li Y. (2020). Scale-Recursive Network with point supervision for crowd scene analysis. Neurocomputing.

[2] Zhong J., Cai W., Lees M., Luo L. (2017). Automatic model construction for the behavior of human crowds. Appl. Soft Comput..

[3] Li J., Wang L., Tang S., Zhang B., Zhang Y. (2016). Risk-based crowd massing early warning approach for public places: A case study in China. Saf. Sci..

[4] Wang J., Jin B., Li J., Chen F., Wang Z., Sun J. (2019). Method for guiding crowd evacuation at exit: The buffer zone. Saf. Sci..

[5] Chen X.-H., Lai J.-H. (2019). Detecting abnormal crowd behaviors based on the div-curl characteristics of flow fields. Pattern Recognit..

[6] Behera S., Dogra D.P., Bandyopadhyay M.K., Roy P.P. (2020). Estimation of linear motion in dense crowd videos using Langevin model. Expert Syst. Appl..

[7] Ma Y., Li L., Zhang H., Chen T. (2017). Experimental study on small group behavior and crowd dynamics in a tall office building evacuation. Phys. A Stat. Mech. Appl..

[8] Shiwakoti N., Gong Y., Shi X., Ye Z. (2015). Examining influence of merging architectural features on pedestrian crowd movement. Saf. Sci..

[9] Karamouzas I., Overmars M. (2011). Simulating and Evaluating the Local Behavior of Small Pedestrian Groups. IEEE Trans. Vis. Comput. Graph..

[10] Xu M., Wu Y., Lv P., Jiang H., Luo M., Ye Y. (2015). miSFM: On combination of Mutual Information and Social Force Model towards simulating crowd evacuation. Neurocomputing.

[11] Zhang X., Yu Q., Yu H. (2018). Physics Inspired Methods for Crowd Video Surveillance and Analysis: A Survey. IEEE Access.

[12] Cong Y., Yuan J., Liu J. (2013). Abnormal event detection in crowded scenes using sparse representation. Pattern Recognit..

[13] Jin Z., Bhanu B. (2015). Analysis-by-synthesis: Pedestrian tracking with crowd simulation models in a multi-camera video network. Comput. Vis. Image Underst..

[14] Fradi H., Eiselein V., Dugelay J.-L., Keller I., Sikora T. (2015). Spatio-temporal crowd density model in a human detection and tracking framework. Signal. Process. Image Commun..

[15] Zhang Y., Chang F., Wang M., Zhang F., Han C. (2018). Auxiliary learning for crowd counting via count-net. Neurocomputing.

[16] Fradi H., Dugelay J.-L. (2015). Towards crowd density-aware video surveillance applications. Inf. Fusion.

[17] Yuan Y., Feng Y., Lu X. (2017). Statistical Hypothesis Detector for Abnormal Event Detection in Crowded Scenes. IEEE Trans. Cybern..

[18] Zhang X., Shu X., He Z. (2019). Crowd panic state detection using entropy of the distribution of enthalpy. Phys. A Stat. Mech. Appl..

[19] Saleh S.A.M., Suandi S.A., Ibrahim H. (2015). Recent survey on crowd density estimation and counting for visual surveillance. Eng. Appl. Artif. Intell..

[20] Fu M., Xu P., Li X., Liu Q., Ye M., Zhu C. (2015). Fast crowd density estimation with convolutional neural networks. Eng. Appl. Artif. Intell..

[21] Shannon C.E. (1948). A mathematical theory of communication. Bell Syst. Tech..

[22] Shannon C.E., Wyner S.A. (1993). Collected Papers.

[23] Tribus M., McIrvine E.C. (1971). Energy and information. Sci. Am..

[24] Wan J., Guo N. (2019). Shannon Entropy in Configuration Space for Ni-Like Isoelectronic Sequence. Entropy.

[25] Nicolis O., Mateu J., Contreras-Reyes J.E. (2020). Wavelet-Based Entropy Measures to Characterize Two-Dimensional Fractional Brownian Fields. Entropy.

[26] Farhan A.K., Al-Saidi N.M.G., Maolood A.T., Nazarimehr F., Hussain I. (2019). Entropy Analysis and Image Encryption Application Based on a New Chaotic System Crossing a Cylinder. Entropy.

[27] Zadeh L.A. (1965). Fuzzy sets. Inf. Control.

[28] (1977). Mamdani Application of Fuzzy Logic to Approximate Reasoning Using Linguistic Synthesis. IEEE Trans. Comput..

[29] Wang W., Tong S. (2019). Observer-Based Adaptive Fuzzy Containment Control for Multiple Uncertain Nonlinear Systems. IEEE Trans. Fuzzy Syst..

[30] Feng S., Chen C.L.P. (2020). Fuzzy Broad Learning System: A Novel Neuro-Fuzzy Model for Regression and Classification. IEEE Trans. Cybern..

[31] Mintz A. (1951). Non-adaptive group behavior. J. Abnorm. Soc. Psychol..

[32] Kelley H.H., Condry J.C., Dahlke A.E., Hill A.H. (1965). Collective behavior in a simulated panic situation. J. Exp. Soc. Psychol..

[33] Helbing D., Molnár P. (1995). Social force model for pedestrian dynamics. Phys. Rev. E.

[34] Helbing D., Farkas I., Molnar P., Vicsek T. (2002). Simulation of Pedestrian Crowds in Normal and Evacuation Situations. Pedestr. Evacuation Dyn..

[35] Helbing D., Farkas I.J., Vicsek T. (2000). Simulating dynamical features of escape panic. Nature.

[36] Farrahi K., Zia K., Sharpanskykh A., Ferscha A., Muchnik L. (2013). Agent Perception Modeling for Movement in Crowds. Proceedings of the 11th International Conference on Practical Applications of Agents and Multi-Agent Systems (PAAMS), Germany, 22–24 May 2013.

[37] Golas A., Narain R., Lin M.C. (2014). Continuum modeling of crowd turbulence. Phys. Rev. E.

[38] Golas A., Narain R., Curtis S., Lin M.C. (2013). Hybrid Long-Range Collision Avoidance for Crowd Simulation. IEEE Trans. Vis. Comput. Graph..

[39] Stephen J.G., Kim S., Lin M., Manocha D. Simulating heterogeneous crowd behaviors using personality trait theory. Proceedings of the 2011 ACM SIGGRAPH/Eurographics Symposium on Computer Animation.

[40] Akopov A.S., Beklaryan L.A. (2015). An agent model of crowd behavior in emergencies. Autom. Remote. Control.

[41] Gu X., Cui J., Zhu Q. (2014). Abnormal crowd behavior detection by using the particle entropy. Optik.

[42] Zhao K., Liu B., Li W., Yu N., Liu Z. Anomaly Detection and Localization: A Novel Two-Phase Framework Based on Trajectory-Level Characteristics. Proceedings of the 2018 IEEE International Conference on Multimedia & Expo. Workshops (ICMEW).

[43] Hao Y., Xu Z., Liu Y., Wang J., Fan J.-L. (2018). Effective Crowd Anomaly Detection Through Spatio-temporal Texture Analysis. Int. J. Autom. Comput..

[44] Zhang X., Zhang Q., Hu S., Guo C., Yu H. (2018). Energy Level-Based Abnormal Crowd Behavior Detection. Sensors.

[45] Davies A., Velastin S., Yin J.H. (1995). Crowd monitoring using image processing. Electron. Commun. Eng. J..

[46] Ma W., Huang L., Liu C. Advanced Local Binary Pattern Descriptors for Crowd Estimation. Proceedings of the 2008 IEEE Pacific-Asia Workshop on Computational Intelligence and Industrial Application.

